# Abolition of seizures following Forel‐H‐tomy for drug‐resistant epilepsy: A case report

**DOI:** 10.1002/epi4.12826

**Published:** 2023-09-25

**Authors:** Shiro Horisawa, Satoru Miyao, Tomokatsu Hori, Kilsoo Kim, Takakazu Kawamata, Takaomi Taira

**Affiliations:** ^1^ Department of Neurosurgery Tokyo Women's Medical University Shinjyuku Japan; ^2^ Department of Neurosurgery TMG Asaka Medical Center Asaka Japan; ^3^ Department of Neurosurgery Moriyama Memorial Hospital Edogawa City Japan

**Keywords:** fields of Forel, pallidothalamic tract, radiofrequency ablation

## Abstract

A 62‐year‐old female experienced an extensive cerebral contusion in the left frontotemporal lobe due to an acute subdural hematoma at the age of 44 years. Six months after the injury, the patient developed epileptic seizures. The seizures were generalized with right cervical rotation and fencing posture. Despite prescriptions for four antiepileptic drugs, partial seizures occurred several times a month and focal to bilateral tonic‐clonic seizures once every 2 months. Video‐electroencephalography showed epileptic discharges in the left frontal lobe. The patient was subsequently referred to our department for palliative surgery. The patient underwent a left Forel‐H‐tomy. The prescription of antiepileptic drugs was not changed, and the patient was seizure free for 1 year. Forel‐H‐tomy, a surgical procedure for intractable epilepsy, was pioneered by Dennosuke Jinnai. Despite its previously reported remarkable efficacy, Forel‐H‐tomy has not been performed for several decades. Nevertheless, it remains a potential alternative treatment option for drug‐resistant epilepsy.

## 1 INTRODUCTION

In the 1960s and 1970s, Dennosuke Jinnai performed Forel‐H‐tomy as a procedure for intractable epilepsy.^1^ Studies investigating various seizure conduction pathways have reported that lesion in Forel's field H can effectively reduce epileptic seizures by blocking seizure impulses.^2^, ^3^, ^4^ However, it is no longer used for this purpose. Instead, the field of Forel (FF), a region located caudal to the thalamus, is a therapeutic target for movement disorders such as Parkinson's disease and dystonia. This is due to its role as a convergent point of the pallidothalamic tract from the globus pallidus interna (GPi) to the thalamus in the basal ganglia‐thalamocortical circuit (BTCC).^5^ Recent studies have highlighted the involvement of the basal ganglia in the pathogenesis of FBTCS, seizure termination, and the network of poststroke epilepsy.^6^, ^7^, ^8^, ^9^ This has led to a growing understanding of the potential therapeutic effects of Forel‐H‐tomy on epilepsy. Our previous report has shown that Forel‐H‐tomy dramatically reduced seizures in two patients with movement disorders and comorbid epilepsy.^10^ In this case report, we describe a patient with drug‐refractory epilepsy of left frontal lobe origin that occurred once a month and was seizure‐free for 1 year following Forel‐H‐tomy. This is the first report of an MRI‐guided Forel‐H‐tomy for epilepsy without a concomitant movement disorders.

## 2 CASE REPORT

**FIGURE 1:**
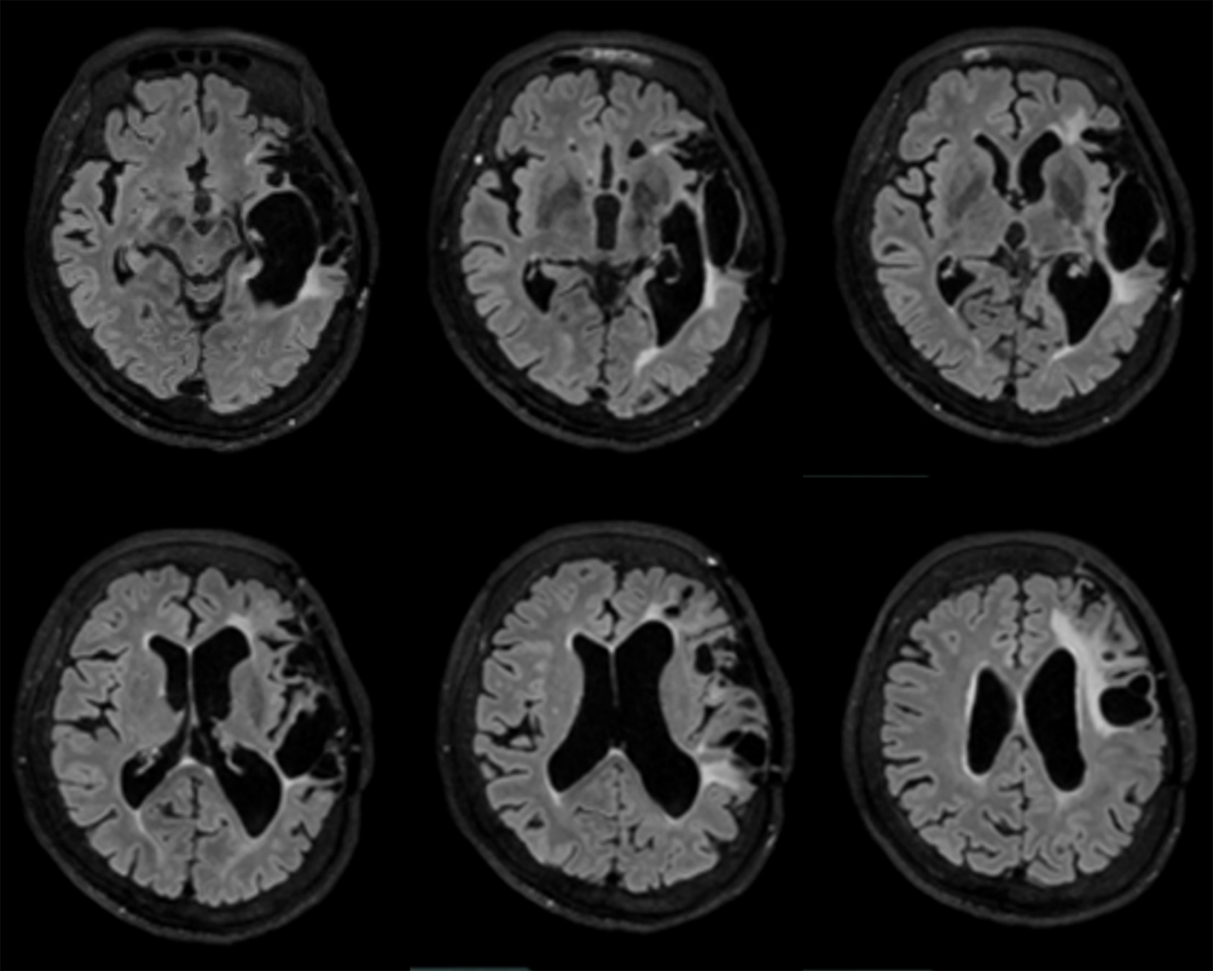
Pre‐operative fluid attenuated inversion recovery images. Left frontotemporal encephalomalacia secondary to severe traumatic brain injury caused by titanium mesh cranioplasty.

**FIGURE 2:**
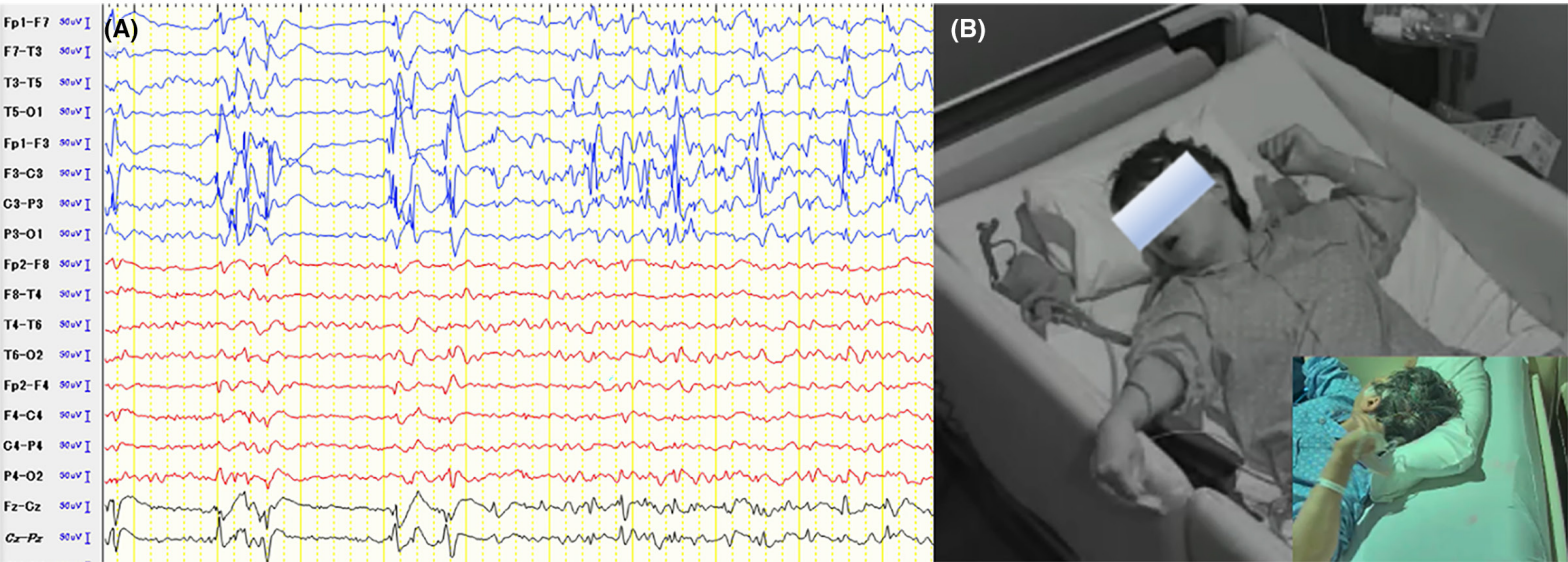
Seizure onset on ictal video electroencephalography (EEG). (A) Scalp EEG shortly after seizure onset showing ongoing ictal activity in the left frontal lobe. (B) Fencing posture during a focal to bilateral tonic‐clonic seizure.

This report presents the case of a 62‐year‐old female who had been diagnosed with left frontal lobe epilepsy 19 years prior. She had sustained a left acute subdural hematoma and a left frontotemporal lobe cerebral contusion in a traffic accident at 44 years of age. The patient underwent a decompression craniotomy and a titanium mesh cranioplasty (Figure 1A). Six months later, the patient developed epileptic seizures with loss of consciousness. The seizures were accompanied by right cervical rotation, and the contralateral arm was abducted at the shoulder and externally rotated and flexed at the elbow. Despite prescriptions for multiple antiepileptic drugs, she continued to experience partial seizures in the right face and right upper extremity once a month, as well as focal to bilateral tonic‐clonic seizures every 2 months. The patient was referred to our department for further surgical treatment. The prescribed antiepileptic drugs were mystan 15 mg, levetiracetam 2000 mg, lacosamide 300 mg, and phenytoin 480 mg. A family member living with the patient reported several partial seizures and one FBTCS per month. Video electroencephalography (EEG) showed an electrographic seizure with onset from the left frontal scalp electrodes, which evolved in frequency and amplitude over the bilateral hemispheres, with focal to bilateral tonic‐clonic seizures manifested by a fencing posture (Figure 2A,B). After the seizure, aphasia symptoms appeared for approximately 1 h. A diagnosis of neocortical epilepsy of left frontal lobe origin was made. The patient was reluctant to have the device implanted, and after obtaining consent from the patient and family, a left Forel‐H‐tomy was performed.

**FIGURE 3:**
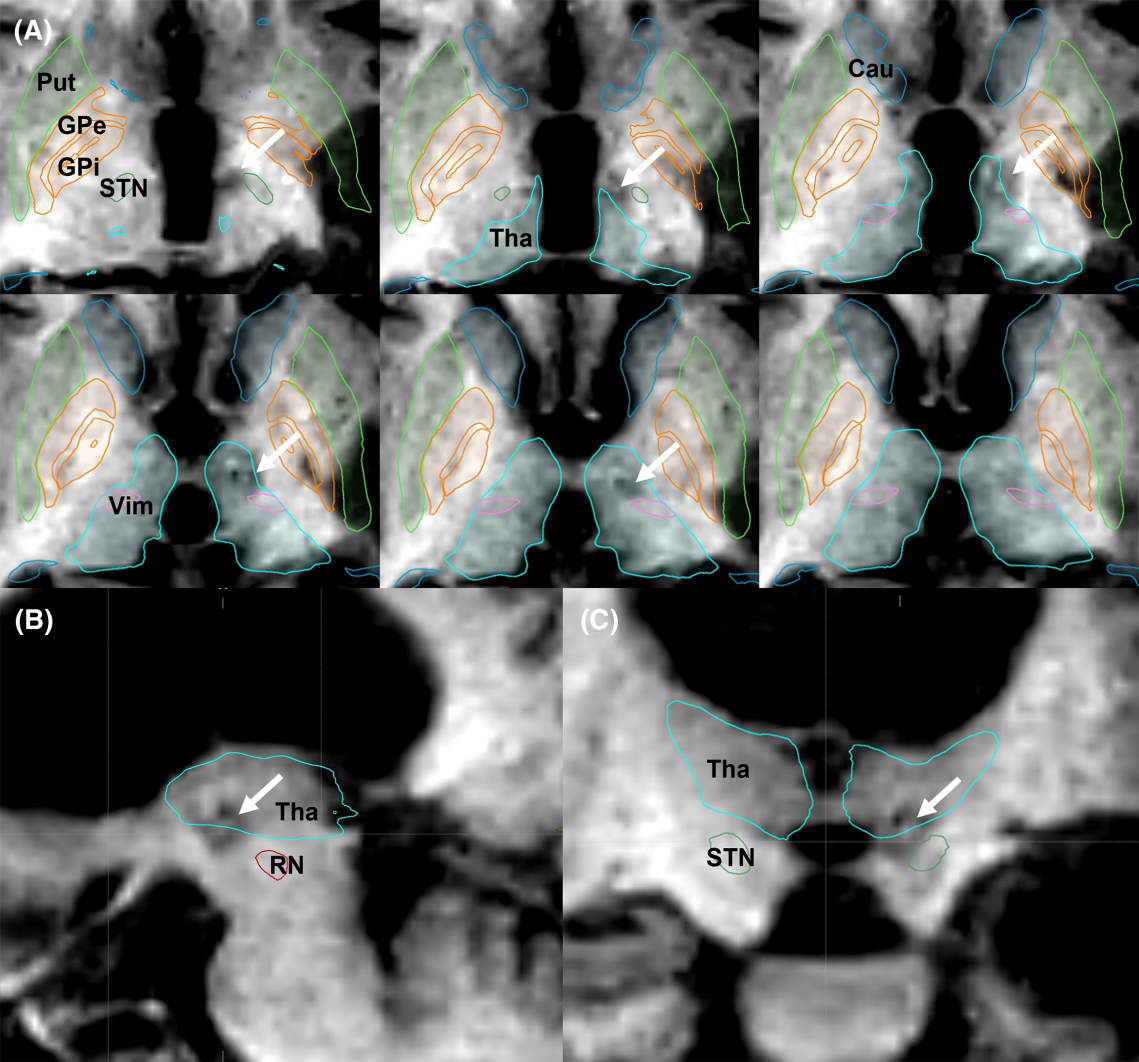
Postoperative magnetic resonance imaging (MRI) with anatomical mapping Brainlab elements. Postoperative T1‐weighted axial (A), sagittal (B), and coronal (C) MRI showing lesion (white arrow) and surrounding structures. Cau, caudate nucleus; GPi, globus pallidus internus; GPe, globus pallidus externus; Put, putamen; RN, red nucleus; STN, subthalamic nucleus; Tha, thalamus; Vim, ventral intermediate nucleus.

The surgery was carried out under local anesthesia without recording the microelectrodes. A parietal lobe approach was chosen due to the challenges presented by extensive atrophy of the left frontal lobe and the presence of titanium mesh cranioplasty, making the frontal lobe approach difficult. Stereotactic planning was performed using the Brainlab Elements software (Brainlab), and the target for Forel‐H‐tomy was located in FF H1, where the ansa lenticularis and lenticular fasciculus merge, corresponding to the thalamic fasciculus on the Morel atlas. The target was positioned at 8.5 mm lateral, 0.5 mm posterior, and 1.5 mm inferior to the midpoint of the anterior commissure‐posterior commissure line. We used a monopolar radiofrequency probe (1.0‐mm diameter tip with an uninsulated length of 4.0 mm) and a Leksell NeuroGenerator (Elekta) to confirm impedance monitoring and induce macrostimulation and coagulation. A single radiofrequency lesion was generated at 70°C for 40 s (Figure 3A–C), resulting in a lesion volume of 25 mm^3^. Postoperatively, the patient experienced temporary weakness in the right upper and lower limbs, which did not interfere with activities of daily living and fully recovered within 3 months. The last follow‐up, conducted 1 year after the surgery, showed no occurrence of epileptic seizures (Engel outcome scale I), including focal seizures or FBTCS, without any alterations to the prescribed antiepileptic drug regimen.

## 3 DISCUSSION

For drug‐resistant epilepsy of left frontal lobe origin, ipsilateral Forel‐H‐tomy resulted in complete resolution of monthly‐onset epileptic seizures during a 1‐year follow‐up period. This suggests the efficacy of Forel‐H‐tomy for refractory epilepsy, which was previously reported by Dennosuke Jinnai. However, this technique was abandoned for an extended period. During the 1970s, there was growing social criticism of lobotomies in Japan, leading to the Japanese Society of Psychiatry and Neurology passing a resolution in 1975 rejecting psychosurgery, which included epilepsy as one of the indications.^11^ Consequently, stereotactic brain surgery for epilepsy, such as Forel‐H‐tomy, was subsequently associated with ‘psychosurgery’ and ceased to be performed.

Jinnai et al. investigated seizure conductive pathways by using multiple electrodes to detect spike waves on the basal ganglia, including the caudate nucleus, globus pallidus, and substantia nigra, and thalamus.^2^ They proposed that convulsive impulses eventually reach the substantia nigra via the globus pallidus, descending and propagating to the spinal cord.^2^, ^3^ They identified the Forel's field H (FF H) as the area through which the fibers connecting the globus pallidus and substantia nigra pass, suggesting that lesioning this area could disrupt seizure conduction pathways and alleviate epilepsy.^12^ Furthermore, Jinnai preferred white matter as a surgical target because of the induction of epileptogenicity by gray matter lesions such as the globus pallidus and substantia nigra.^13^ Additionally, the midbrain contains various neural nuclei with diverse functions, making it another reason to avoid targeting the substantia nigra.^13^ They also noted that the FF H concentrates many fibers in a relatively small area and that beneficial effects can be achieved with a small lesion.^13^ The rationale behind Forel‐H‐tomy for epilepsy is to interrupt the pathway of epileptic discharge (descending impulse) and elevate the seizure threshold, based on experimental animal work.^3^, ^14^

Jinnai et al. reported the efficacy of Forel‐H‐tomy in 64 patients with intractable epilepsy, including generalized convulsion, focal convulsion, and Lennox–Gastaut syndrome.^15^ In well‐placed lesions (unilateral or bilateral), significant seizure reduction (diminished or abolished) was confirmed.^15^ In obvious asymmetrical seizure manifestation confirmed symptomatically or electroencephalographically, Forel‐H‐tomy on the ipsilateral side of the seizure origin could achieve sufficient seizure control.^1^ The lesion coordinates of the Forel‐H‐tomy performed by Jinnai et al. were reported as 2 mm posterior, 4.5–5 mm inferior to the AC‐PC midpoint, and 4 mm lateral to the third ventricular wall.^1^ However, it is unclear whether the lesion was correctly located in the FF H, as CT and MRI were inconclusive in confirming the lesion site. It is possible that the lesion extended to the thalamus, such as the centromedian and ventrolateral nuclei, or that it also involved the cerebellothalamic tract, which has been reported to be effective for epilepsy, possibly due to lesion encroachment on the neighboring structures.^16^, ^17^, ^18^ The target position of Jinnai et al. is slightly lower and posterior to ours. In our patient, because we used a parietal approach, the proximal part of the lesion by the 4 mm electrode length included the area 1–2 mm posterior to the midpoint of the AC‐PC. In addition, a target 4.5–5 mm below the AC‐PC line in this patient was located in the midbrain region. Therefore, we decided to target 1.5 mm below the AC‐PC line to reduce the surgical risk. The result of the present case was consistent with Jinnai's concept of Forel‐H‐tomy of significant seizure reduction after a tiny lesion at the FF H.

Animal experiments and clinical studies in humans have indicated that the BTCC is involved in the pathophysiology of epilepsy.^9^, ^19^ Specifically, dysfunction of the BTCC is thought to be involved in the mechanism of secondary generalization. He et al. investigated the functional abnormality of BTCC in relation to vulnerability to FBTCS in patients with temporal lobe epilepsy.^9^ In their study, they found that striatal inhibition of the thalamus from the GPi could lead to an under‐suppressed thalamus (imbalance between basal ganglia inhibition and thalamus synchronization), which in turn may account for the greater vulnerability of these patients to secondary seizure generalization.^9^ This study discusses the theoretical background of the treatment mechanism of surgical intervention with BTCC for epilepsy. Several studies have reported that deep brain stimulation (DBS) of the GPi and the subthalamic nucleus (STN), which constitute the BTCC, suppresses epileptic seizures. The most commonly performed surgery for BTCC is available for movement disorders such as Parkinson's disease and dystonia. The GPi, the major output structure of the basal ganglia, is available as a target for ablation and DBS. Taylor et al. reported that GPi‐DBS for Parkinson's disease controlled seizures that were catatonic with left head turning and rigidity.^20^ Interestingly, in this report, EEG revealed multiple right focal seizures with secondary generalization and the seizures were completely controlled by ipsilateral right‐sided GPi‐DBS alone.^20^ Handforth et al. performed STN‐DBS on two patients with drug‐resistant epilepsy (post‐traumatic and post‐encephalitic epilepsy) and reported seizure‐frequency reductions of 44% and 29%, respectively.^21^ Chabardes et al. performed STN‐DBS for various drug‐resistant epilepsies and reported a seizure frequency reduction of 64%.^22^ Elhadd et al. reported a case of dystonic tremor with juvenile myoclonic epilepsy treated with DBS targeting the ventral intermediate nucleus and zona incerta (ZI).^23^ The stimulation not only improved dystonic tremor but also dramatically decreased seizure frequency from two to three seizures per week before surgery to two seizures in 3 years following surgery.^23^ ZI is the gray matter structure situated between the lenticular fasciculus and FF H1. Costimulation of pallidal projections to the thalamus may be responsible for seizure reduction. In our previous studies, two patients with movement disorders and comorbid epilepsy experienced significant seizure reduction after radiofrequency ablation of the pallidothalamic tract in the FF.^10^ These results suggest the efficacy of Forel‐H‐tomy for epilepsy, as reported earlier by Dennosuke Jinnai, and led us to initiate research on Forel‐H‐tomy for epilepsy.

In the present case, there are several possible mechanisms for improving epilepsy. The pallidothalamic tract projects to the CM and VL nuclei of the thalamus via the FF H.^5^ A lesion at FF H may improve epilepsy by a mechanism similar to CM/VL‐DBS. In addition, the FF H area is lateral to the mammillothalamic tracts (MTTs). The mammillothalamic tract projects to the anterior thalamic nucleus, an effective DBS target for epilepsy. Schaper et al. reported that the anterior thalamus‐MMT junction is the optimal stimulation site.^24^ Schaper's study suggested that the MTT, which forms the Papez circuit, may be an effective target for the suppression of epilepsy. In this case, the relationship between MTT and lesion could not be clearly depicted.

The present study suggests that Forel‐H‐tomy, which does not require device implantation, can be an alternative treatment option for drug‐resistant epilepsy. Larger sample sizes are needed to further investigate the efficacy of the Forel‐H‐tomy for epilepsy.

## AUTHOR CONTRIBUTIONS

SH, SM, and KK were involved in data acquisition. SH, TH, TK, and TT contributed to the study's design. SH conducted data analysis, drafted the text, and prepared the figures.

## CONFLICT OF INTEREST STATEMENT

The authors declare no conflict of interest. We confirm that we have read the Journal's position on issues involved in ethical publication and affirm that this report is consistent with those guidelines.

## ETHICS STATEMENT

The study was approved by the Institutional Review Board of the Tokyo Women's Medical University (2021‐0009).

## PATIENT CONSENT STATEMENT

Written informed consent was obtained from the patient. We confirm that we have read the Journal's position on issues involved in ethical publication and affirm that this report is consistent with those guidelines.
